# Supplementary material for: An Environmental and Nutritional Evaluation of School Food Menus in Bahia, Brazil That Contribute to Local Public Policy to Promote Sustainability
Source: Nutrients. 2022 Apr 6;14(7):1519. doi: 10.3390/nu14071519 (PMC9003184; doi:10.3390/nu14071519)
Supplement: Supplementary file 1 [file nutrients-14-01519-s001.zip › nutrients-1596503-supplementary.pdf]

Table S1: Nutritional targets for the menus according to the age group.

Nutrient	Age groups							
	7 - 11 months	1 - 3 year	4 - 5 year	6 - 10 year	11 - 15 year	16 - 18 year	19 - 30 year	31 - 60 year
**Energy, kcal**	450	700	950	450	650	750	680	650
**Carbohydrates, g**	73.1	114.9	154.4	73.1	105.6	121.8	110.5	105.6
**Protein, g**	14.0	21.9	29.7	14	20.3	23.4	21.3	20.3
**Total fat, g**	11.3	17.5	23.8	11.3	16.3	18.8	17	16.3
**Saturated fat, g**	5.0	7.8	10.6	5.0	7.2	8.3	7.6	7.2
**Cholesterol, mg**	210	210	210	90	90	90	90	90
**Fiber, mg**		13.3	17.5	8.0	9.0	9.6	9.5	8.5
**Added sugar, g**	0	0	23.8	11.3	16.3	18.8	17	16.3
**Sodium, mg**	1400	1400	1400	600	600	600	600	600
**Calcium, mg**	189	350	560	315	390	390	300	330
**Iron, mg**	7.7	4.9	7.0	2.7	3.2	3.9	3.9	3.2
**Magnesium, mg**	54	56	91	56	95	116	107	111
**Zinc, mg**	2.1	2.1	3.5	2.0	2.7	3	2.9	2.9
**Vitamin A, mcg**	350	210	280	150	210	240	240	240
**Vitamin C, mg**	35	12	19	11	18	21	26	26
**Vitamin B12, mcg**	0.35	0.63	0.84	0.54	0.54	0.72	0.72	0.72

Table S2: Total GHGE per year in Kg of CO_2e of school food menus according to the age group and percentage of menus being sustainable. Total number of students served in 200 scholar days was 32,000.

Year	Group 1		Group 2		% of menus being sustainable
	Conventional	Sustainable	Conventional	Sustainable	
**2018**	400.44	0	241.95	0	0%
**2019/01**	160.18	20.00	96.78	10.56	20%
**2019/02**	120.13	40.00	72.59	21.12	40%
**2020/01**	80.09	60.00	48.39	31.68	60%
**2020/02**	40.04	80.00	24.20	42.24	80%
**2021**	80.09	160.00	48.39	84.48	80%

Table S3: List of ingredients and *per capita* amount of conventional and sustainable school food menus.

Conventional Food Menu				
**Recipe number**	**Food recipe**	**Ingredients**	**Unit (g/ml)**	***Per capita* (g/ml)**
1	Latte	Coffee	g	5
		Milk Powder	g	20
		Tap Water	ml	200
		Sugar	g	15
2	Banana	Banana	g	75
3	Mango	Mango	g	30
4	Papaya	Papaya	g	30
5	Watermelon	Watermelon	g	100
6	Apple	Apple	g	50
7	Guava	Guava	g	50
8	Mango Juice	Tap Water	ml	200
		Mango	g	50
		Sugar	g	15
9	Acerola Juice	Tap Water	ml	200
		Acerola	g	50
		Sugar	g	15
10	Guava Juice	Tap Water	ml	200
		Guava	g	50
		Sugar	g	15
12	Banana and Apple Smoothie	Banana	g	50
		Apple	g	50
		Peanut Milk	ml	30
		Tap Water	ml	120
		Sugar	g	20
12	Porridge	Milk Powder	g	20
		Tap Water	ml	200
		Multigrain Oat Cereal	g	20
13	Sweetcorn Pudding	Flaked Cornflour	g	20
		Milk Powder	g	20
		Sugar	g	15
		Coconut Milk	ml	3
		Tap water	ml	200
14	Sweet Rice Pudding	White Rice	g	30
		Milk Powder	g	20
		Coconut Milk	ml	3
		Fresh Coconut	g	20
		Sugar	g	15
		Clove	g	1
		Tap Water	ml	200
15	Cornmeal Couscous	Flaked Cornflour	g	30
		Salt	g	1
		Margarine	g	3
		Tap Water	ml	200
16	Tapioca Crepe	Tapioca starch	g	40
		Salt	g	1
17	Brazilian Style Carrot Cake	Wheat Flour	g	50
		Sugar	g	15
		Egg	g	10
		Baking Powder	g	30
		Carrot	g	10
		Soybean Oil	ml	4
18	Bread	Bread	g	50
19	Coconut Cookies	Wheat Flour	g	50
		Fresh Coconut	g	20
		Sugar	g	15
		Butter	g	30
20	Cream Cracker	Cracker	g	40
22	Rice	Onion	g	8
		Garlic	g	1
		Soybean Oil	ml	4
		Salt	g	1
		Tap Water	ml	60
		Parboiled Rice	g	30

Conventional Food Menu				
**Recipe number**	**Food recipe**	**Ingredients**	**Unit (g/ml)**	***Per capita* (g/ml)**
23	Beans	Pinto Beans	g	20
		Garlic	g	1
		Soybean Oil	ml	4
		Tap Water	ml	60
		Salt	g	1
		Onion	g	8
24	Muleteer Beans with Beef Jerky	Black Eyed Beans	g	20
		Garlic	g	1
		Soybean Oil	ml	4
		Onion	g	8
		Tomato	g	10
		Bell Pepper	g	3
		Coriander	g	1.5
		Parsley	g	1
		Bay Leaves	g	1
		Salt	g	1
		Chives	g	1
		Tap Water	ml	60
		Jerky	g	20
		Cassava Flour	g	15
25	Pasta with Sardine and Tomato Sauce	Spaghetti Pasta	g	30
		Canned Sardines	g	20
		Soybean Oil	ml	4
		Garlic	g	1
		Onion	g	8
		Bell Pepper	g	3
		Parsley	g	1
		Oregano	g	1
		Salt	g	1
		Chives	g	1
		Coriander	g	1.5
		Tap Water	ml	300
		Tomato	g	10
		Canned Tomato Sauce	g	10
26	Pasta with Tomato Sauce	Spaghetti pasta	g	30
		Soybean Oil	ml	4
		Garlic	g	1
		Onion	g	8
		Tomato	g	10
		Bell Pepper	g	3
		Parsley	g	1
		Chives	g	1
		Tap water	ml	300
		Oregano	g	1
		Salt	g	1
		Canned Tomato Sauce	g	10
27	Beef Bolognese Pasta	Pasta	g	30
		Soybean Oil	ml	4
		Garlic	g	1
		Onion	g	8
		Canned Tomato Sauce	g	10
		Tomato	g	10
		Bell Pepper	g	3
		Parsley	g	1
		Chives	g	1
		Coriander	g	1.5
		Tap Water	ml	300
		Oregano	g	1
		Salt	g	1
		Minced Beef	g	30

Conventional Food Menu				
**Recipe number**	**Food recipe**	**Ingredients**	**Unit (g/ml)**	***Per capita* (g/ml)**
**28**	Beef Stew with Potato and Carrot	Diced beef	g	30
		Onion	g	8
		Garlic	g	1
		Soybean Oil	ml	4
		Potato	g	25
		Carrot	g	10
		Canned Tomato Sauce	g	10
		Tomato	g	10
		Bell Pepper	g	3
		Parsley	g	1
		Chives	g	1
		Oregano	g	1
		Salt	g	1
		Black Pepper	g	1
**29**	Fish Fillet with Potato	White Fish Filet	g	80
		Garlic	g	1
		Onion	g	8
		Soybean Oil	g	4
		Potato	g	25
		Cocunut Milk	ml	3
		Tomato	g	10
		Bell Pepper	g	3
		Parsley	g	1
		Chives	g	1
		Oregano	g	1
		Salt	g	1
		Black Pepper	g	1
**30**	Chicken	Chicken Thigh	g	80
		Canned Tomato Sauce	g	10
		Garlic	g	1
		Onion	g	8
		Soybean Oil	ml	4
		Tomato	g	10
		Bell Pepper	g	3
		Parsley	g	1
		Chives	g	1
		Coriander	g	1.5
		Oregano	g	1
		Salt	g	1
		Black Pepper	g	1
		Vinager	g	5
		Lime	g	2
**31**	Diced Chicken Breast	Chicken Breast	g	50
		Canned Tomato Sauce	g	10
		Garlic	g	1
		Onion	g	8
		Soybean Oil	ml	4
		Tomato	g	10
		Bell Pepper	g	3
		Parsley	g	1
		Chives	g	1
		Oregano	g	1
		Salt	g	1
		Black Pepper	g	1
		Vinager	g	5
		Lime	g	2
**32**	Cassava with Pulled Beef	Cassava	g	60
		Minced Beef	g	77
		Garlic	g	1
		Onion	g	8
		Soybean Oil	ml	4
		Tomato	g	10
		Bell Pepper	g	3
		Parsley	g	1
		Chives	g	1
		Oregano	g	1
		Salt	g	1
		Tap Water	ml	120
		Spicy Salt	g	2
		Black Pepper	g	1

Conventional Food Menu				
**Recipe number**	**Food recipe**	**Ingredients**	**Unit (g/ml)**	***Per capita* (g/ml)**
**33**	Mashed Potato	Potato	g	25
		Salt	g	1
		Milk Powder	g	20
		Margarine	g	3
		Soybean Oil	ml	4
		Onion	g	8
		Garlic	g	1
		Tap Water	ml	200
**34**	Eggs Omelet	Egg	g	50
		Potato	g	25
		Carrot	g	10
		Salt	g	1
		Tomato	g	10
		Wheat Flour	g	50
		Butter	g	3
**35**	"Couscous with Josefina"	Flaked Cornflour	g	30
		Tap Water	ml	200
		Salt	g	1
		Smoked Sausage	g	10
		Onion	g	8
		Soybean Oil	ml	4
		Tomato	g	10
		Bell Pepper	g	3
		Parsley	g	1
		Chives	g	1
**36**	Vegetable and Chicken Soup	Chicken	g	50
		Pasta	g	20
		Salt	g	1
		Pumpkin	g	25
		Garlic	g	1
		Onion	g	8
		Carrot	g	10
		Potato	g	25
		Coriander	g	1.5
		Tap Water	ml	200
		Tomato	g	10
		Soybean Oil	ml	4
**37**	Sweet Potato	Sweet Potato	g	25
**38**	Carrot	Carrot	g	10
**39**	Bread with Margarine	Bread	g	50
		Margarine	g	3
**40**	Sweet Potato with Egg	Sweet Potato	g	25
		Salt	g	1
		Egg	g	50
**41**	Braised Kale	Kale	g	5
		Garlic	g	1
		Soya oil	ml	4
		Salt	g	1
**42**	Hot Chocolate	Chocolate Powder	g	150
		Tap water	ml	250
		Milk Powder	g	20
**43**	Bread with Fried Egg	Bread	g	50
		Egg (fried)	g	50
		Soybean Oil	ml	4
**44**	Guava Jam	Guava	g	28
		Tap Water	ml	6
		Sugar	g	17
		Passion Fruit	ml	14
		Lime	ml	0.14
**45**	Yogurt (any flavour)	Yogurt	g	200
**46**	Crackers with Guava Jam	Guava Jam	g	50
		Crackers	g	163

Sustainable Food Menu

Recipe number	Food recipe	Ingredients	Unit (g/ml)	*Per capita* (g/ml)
1	Peanut Milk	Peanuts	g	30
		Tap Water	ml	200
2	Banana	Banana	g	75
3	Mango	Mango	g	30
4	Papaya	Papaya	g	30
5	Watermelon	Watermelon	g	100
6	Apple	Apple	g	50
7	Mango Juice	Tap Water	ml	200
		Mango	g	50
		Sugar	g	15
8	Acerola Juice	Tap Water	ml	200
		Acerola	g	50
		Sugar	g	15
9	Guava Juice	Tap Water	ml	200
		Guava	g	50
		Sugar	g	15
11	Papaya and Apple Smoothie	Papaya	g	50
		Apple	g	50
		Peanut Milk	ml	30
		Tap Water	ml	120
		Sugar	g	20
12	Banana and Apple Smoothie	Banana	g	50
		Apple	g	50
		Peanut Milk	ml	30
		Tap Water	ml	120
		Sugar	g	20
13	Banana and Papaya Smoothie	Papaya	g	50
		Banana	g	50
		Peanut Milk	ml	30
		Tap Water	ml	120
		Sugar	g	20
14	Sweetcorn Coconut Pudding	Coconut Milk	ml	3
		Peanut Milk	ml	30
		Tap Water	ml	120
		Hominy	g	20
		Sugar	g	15
15	Cornmeal Porridge	Peanut Milk	ml	30
		Tap Water	ml	120
		Fine Cornmeal	g	20
		Sugar	g	15
16	Sweet Rice Pudding	Rice	g	30
		Tap Water	ml	60
		Sugar	g	15
		Coconut Milk	ml	3
		Peanut Milk	ml	30
		Cinnamon Sticks	g	1
		Lemon Zests	g	1
17	Hotdog Bread	Hotdog bread	g	50
18	Soya Mince Sandwich	Soya Protein Mince	g	40
		Canned Tomato Sauce	g	95
		Tap Water	ml	240
		Garlic	g	1
		Onion	g	8
		Carrot	g	20
		Soybean Oil	ml	6
		Coriander	g	2
		Tomato	g	10
		Oregano	g	0
		Salt	g	1
		Bread	g	50
19	Cream Cracker	Cracker	g	17
20	Peanut Butter Candy	Peanut	g	30
		Sugar	g	10
		Tap Water	ml	1
		Salt	g	1
		Soybean Oil	ml	2
21	Rice	Onion	g	8
		Garlic	g	1
		Soybean Oil	ml	4
		Salt	g	1
		Tap Water	ml	200
		Parboiled rice	g	30

Sustainable Food Menu

Recipe number	Food recipe	Ingredients	Unit (g/ml)	*Per capita* (g/ml)
22	Beans	Pinto beans	g	20
		Garlic	g	1
		Onion	g	8
		Tap Water	ml	60
		Salt	g	1
		Soybean Oil	ml	4
		Onion	g	8
23	Vegetable Couscous	Flaked Cornflour	g	30
		Carrot	g	20
		Soya Protein Mince	g	10
		Tomato	g	10
		Bell Peper	g	3
		Onion	g	8
		Garlic	g	1
		Canned Tomato Sauce	g	10
		Soybean Oil	ml	4
		Tap Water	ml	60
		Salt	g	1
		Coriander	g	2
24	Rice and Black-Eyed Beans with Soya Mince	Rice	g	30
		Black-Eyed Beans	g	20
		Carrot	g	20
		Soya Protein Mince	g	10
		Pumpkin	g	25
		Onion	g	8
		Tomato	g	10
		Garlic	g	1
		Soybean Oil	ml	4
		Kale	g	5
		Coriander	g	2
		Salt	g	1
		Tap Water	ml	120
25	Black Beans and Vegetables cassarole	Black Beans	g	15
		Onion	g	8
		Garlic	g	1
		Soybean Oil	ml	4
		Tomato	g	10
		Soya Protein Mince	g	12
		Carrot	g	20
		Potato	g	25
		Sweet Potato	g	50
		Fresh Coconut	g	20
		Beetroot	g	15
		Coriander	g	2
		Salt	g	1
		Bay leaves	g	1
		Tap Water	ml	70
26	Beans and vegetables soup	Pinto Beans	g	20
		Carrot	g	20
		Pumpkin	g	25
		Potato	g	25
		Onion	g	8
		Garlic	g	1
		Soybean Oil	ml	4
		Coriander	g	2
		Salt	g	1
		Chives	g	1
		Tap Water	ml	60
27	Soya Mince Bolongnese Pasta	Pasta	g	30
		Salt	g	1
		Soya Protein Mince	g	40
		Canned Tomato Sauce	g	10
		Garlic	g	1
		Onion	g	8
		Carrot	g	20
		Tap Water	ml	380
		Soybean Oil	ml	4
		Tomato	g	10

Sustainable Food Menu				
**Recipe number**	**Food recipe**	**Ingredients**	**Unit (g/ml)**	***Per capita* (g/ml)**
**28**	Okra and Pumpkin Soya Chunks cassarole	Pumpkin	g	25
		Okra	g	20
		Salt	g	1
		Soybean Oil	ml	4
		Onion	g	8
		Tap Water	ml	80
		Garlic	g	1
		Soya Protein Chunks	g	10
**29**	Plant-based Shepherd's Pie	Soya Protein Chunks	g	10
		Potato	g	25
		Onion	g	8
		Garlic	g	1
		Canned Tomato Sauce	g	11
		Tomato	g	10
		Tap Water	ml	80
		Salt	g	1
		Soybean Oil	ml	4
**30**	Vegetables and Soya Chunks Cassarole	Soya Protein Chunks	g	8
		Canned Tomato Sauce	g	5
		Onion	g	8
		Chives	g	1
		Parsley	g	1
		Potato	g	20
		Carrot	g	15
**31**	Vegetable Soup with Soya Mince	Pasta	g	20
		Salt	g	1
		Pumpkin	g	25
		Garlic	g	1
		Onion	g	8
		Carrot	g	20
		Potato	g	25
		Coriander	g	2
		Tomato	g	10
		Tap Water	ml	340
		Soybean Oil	ml	4
		Soya Protein Chunks	g	10
**32**	Vegetable Couscous	Flaked corn flour	g	40
		Carrot	g	12
		Soya Protein Mince	g	10
		Tomato	g	30
		Bell peper	g	5
		Onion	g	12
		Garlic	g	2
		Canned Tomato Sauce	g	11
		Soybean Oil	ml	6
		Salt	g	1
		Coriander	g	6
		Tap Water	ml	40
**33**	Cassava	Cassava	g	60
**34**	Potato with Carrot	Potato	g	25
		Carrot	g	10
**35**	Lettuce salad	Lettuce	g	5
**36**	Lettuce and Tomato Salad	Lettuce	g	5
		Tomato	g	10
**37**	Cassava Flour	Cassava Flour	g	15
**38**	Coconut Cake	Wheat Flour	g	2
		Sugar	g	1
		Grated Cocunut	g	0
		Soybean Oil	ml	1
		Coconut Milk	ml	1
		Tap Water	ml	1
		Baking Powder	g	1
